# Validity and reliability of Chinese version of Adult Carer Quality of Life questionnaire (AC-QoL) in family caregivers of stroke survivors

**DOI:** 10.1371/journal.pone.0186680

**Published:** 2017-11-13

**Authors:** Yongxia Mei, Beilei Lin, Yingshuang Li, Chunge Ding, Zhenxiang Zhang

**Affiliations:** 1 School of Nursing, Zhengzhou University, Zhengzhou, Henan Province, People’s Republic of China; 2 First Affiliated Hospital of Zhengzhou University, Zhengzhou, Henan Province, People’s Republic of China; 3 School of Nursing, University of North Carolina at Chapel Hill, Chapel Hill, North Carolina, Unites States of America; La Trobe University, AUSTRALIA

## Abstract

The Adult Carer Quality of Life questionnaire (AC-QoL) is a reliable and valid instrument used to assess the quality of life (QoL) of adult family caregivers. We explored the psychometric properties and tested the reliability and validity of a Chinese version of the AC-QoL with reliability and validity testing in 409 Chinese stroke caregivers. We used item-total correlation and extreme group comparison to do item analysis. To evaluate its reliability, we used a test-retest reliability approach, intraclass correlation coefficient (ICC), together with Cronbach’s alpha and model-based internal consistency index; to evaluate its validity, we used scale content validity, confirmatory factor analysis (CFA) and exploratory factor analysis (EFA) via principal component analysis with varimax rotation. We found that the CFA did not in fact confirm the original factor model and our EFA yielded a 31-item measure with a five-factor model. In conclusions, although some items performed differently in our analysis of the original English language version and our Chinese language version, our translated AC-QoL is a reliable and valid tool which can be used to assess the quality of life of stroke caregivers in mainland China. Chinese version AC-QoL is a comprehensive and good measurement to understand caregivers and has the potential to be a screening tool to assess QoL of caregiver.

## Introduction

According to the American Heart Association, on average, a stroke occurs every 40 seconds and a stroke fatality occurs every 4 minutes [1]. In the United Kingdom (UK), stroke is a major cause of morbidity, the third-most-common cause of death for women in England and Wales, and the fourth-most-common cause of death in men [2]. In China, stroke is the most frequent cause of death and the annual incidence rate of stroke is 8.7 per 1000, resulting in costs in excess of more than $100 billion [3,4]. About 75% of Chinese stroke survivors have at least some form of disability and experience difficulty in completing day-to-day chores [5]. More than 80% of disabled stroke patients need long-term care from family caregivers [6]. Caring for patients with stroke results in burden and emotional distress, as well as hope and positive outcomes [7–9], all of which affect the QoL of their caregivers [10,11]. QoL is regarded as an established marker of biopsychosocial health and an increasingly-used measure of caregivers’ health [11,12]. Reports from the literature indicate that the QoL of caregivers can affect the physical outcomes and QoL of patients [13,14]. QoL of Chinese caregivers has been found to be poor in both community and hospital setting (in the short term as well as the long term care contexts), suggesting that these caregivers need support from health providers [15–18]. Consequently, promoting the health of stroke patients may require acknowledging and ensuring the QoL of their caregivers.

The World Health Organization (WHO) defines QoL as a broad multidimensional concept which includes subjective evaluations of both the positive and the negative aspects of life [19]. We focus here on health related QoL, which, according to the National Center for Chronic Disease Prevention and Health Promotion, includes perceptions of both physical and mental health, and their correlates on the individual level [20]. Researchers have used QoL (in the stress-coping framework of Lazarus and Folkman) as the outcome of the process of coping with stressor for caregivers when using the the stress-coping framework of Lazarus and Folkman to explain [21, 22]. White et al. [23] developed a model to evaluate the QoL of family caregivers of stroke survivors in the context of the caregiving situation, the caregiver’s characteristics, and characteristics of the environment, including the balance of positive and negative effects of caregiving. Reports from the literature indicate that the QoL of caregivers for patients with cancer was multidimensional, including psychological, social, mental and physical and behavioral components [22, 24]. However, the QoL of caregivers for patients with stroke has not been studied widely in China, possibly due to the lack of a proper assessment tool.

There are some evidence-based instruments which can be used to assess QoL, most of which are general in nature to facilitate use with different types of subjects. The Short Form-36 Healthy Survey (SF-36) is a tool which previous researchers have used to measure the QoL of healthy people, patients [25], and caregivers for patients with stroke [26]. Researchers have also employed a simplified version of the SF-36, the 12-item Short Form Health Survey [27, 28]. Jeong et al. [29] and Chuluunbaatar et al. [30] have used the WHO Quality of Life instrument (WHOQOL) to measure the QoL of stroke caregivers. These instruments demonstrate good reliability and validity in both English-language forms and Chinese versions, but they are general tools which focus more on the negative (not the positive) aspects of QoL, and are not specifically intended for caregivers of stroke patients [11]. According to Joseph et al. [31], the developers of the AC-QoL, the influences on caregivers may be more wide ranging and affect the caregivers’ entire QoL. More general instruments thus may fail to evaluate domains of particular importance to family caregivers, such as personal growth and stress due to caring.

The AC-QoL is a tool designed specifically to assess the QoL of caregivers, developed by Joseph et al. [31] using an initial panel of 385 caregivers from the UK and principal components analysis to test an set of 100 questions; as a result of their testing. They reduced the number of questions to 40 across eight domains (support for caring, caring choice, caring stress, financial implications, personal growth, sense of value, ability to care and carer satisfaction). The AC-QoL’s internal consistency/reliability is 0.94, indicating that the AC-QoL is a useful tool to assess the QoL of adult caregivers[31]. Brand et al. [11] used the AC-QoL to explore the relationship between social support, benefit finding and QoL in caregivers of patients with mental health difficulties and physical health problems, and reported a Cronbach’s alpha of 0.93 for the AC-QoL. Parker et al. [32] and Brand et al. [33] used the AC-QoL to evaluate the effects of their interventions (Parker et al. peer-support and a peer staffing model for residential mental health rehabilitation; Brand et al. benefit finding through writing) on caregivers of people with mental and physical disabilities. To the best of our knowledge, the AC-QoL has not been used in China and there are no other tools specific to stroke caregivers’ QoL. However, due to the differences in culture and language between the UK and China, we felt it essential to develop a Chinese version AC-QoL so that we could assess the QoL of Chinese caregivers. Since the psychometric properties of AC-QoL also have never been tested in stroke caregivers, to provide preliminary support for the use of the AC-QoL in cross-culture stroke caregiver research, our purpose in this study was two-fold: 1) to translate the English AC-QoL into Chinese; and, 2) to test the reliability and validity of AC-QoL in Chinese stroke caregivers.

## Methods

### Participants

We used a cross-sectional study design with a convenience sampling method. We estimated our desired sample size according to the ratio of subjects-to-items (5–10 subjects per item) [34]. A total of 441 caregivers of patients with stroke from 5 hospitals in Zhengzhou, China, participated in this study. We used the following inclusion criteria for family caregivers: (1) the caregivers participants were the primary (non-professional and unpaid) caregivers; (2) the caregivers were adults 18 years old or older; (3) the caregivers could be contacted by telephone after the discharge of the patient; (4) the caregivers had no obvious cognitive or language disabilities; and, (5) the caregivers were willing to participate in the survey. Of our initial study population, 32 caregivers did not complete the questionnaire. We therefore conducted our analyses on the data provided by 409 caregivers who completed the questionnaire. There was no missing required information. The valid return rate was 92.47%.

### Measures

#### Adult carer quality of life questionnaire

We used the original version of the AC-QoL, a 40-item instrument designed to assess the QoL of adult caregivers [31]. The majority of the AC-QoL items are scored from 0 (never) to 3 (always), but items 6–16, 19, 37 and 38 are scored in reverse. The total possible score is 120, with a higher score indicating a better QoL. Joseph et al. demonstrated that the instrument has adequate internal consistency reliability, with an internal consistency value for the overall AC-QoL of 0.94 and internal consistency values for the eight subscales ranging from 0.78 to 0.89 [31].

### Translation procedure

We conducted a forward-back-translation procedure for the Chinese version of the AC-QoL following the cross-cultural study protocol of Cao et al. [35]. Initially, one translator from Zhengzhou University with a background in chronic care and a second translator (a graduate in nursing science) translated the English version into Chinese. Our research team and the two translators (after discussion) then resolved differences between the two Chinese versions. A Chinese professor of English linguistics with more than 3 years’ experience in English speaking countries, and a second graduate student in nursing science in the UK translated the Chinese version back into English. Finally a Chinese professor in nursing science (a graduate of a university in the United States [US]) and our research team compared the backwards translation with the original English version, provided opinions and confirmed the conceptual and literal equivalence of the Chinese version. We then pre-tested the instrument on 30 stroke caregivers and found that they could understand the items easily and that they required an average of 20 minutes to finish the questionnaire.

### Data collection

We invited 10 experts to evaluate the content validity of the Chinese AC-QoL. These experts included two professors expert in stroke care, two professors with experience in mental health, a professor and an associate professor with experience in questionnaire developments, a professor and an associate professor in caregiver research, and a director nurse and an assistant director nurse in clinical nursing. We used the item content validity index (I-CVI) and scale content validity index (S-CVI) to evaluate the content validity, using a four-point scale ranging from 1 (not relevant) to 4 (highly relevant) [36].

We recruited and trained four graduate students in nursing science as research assistants before the survey to ensure that they were familiar with the study procedure and skilled with the questionnaire. The research assistants informed patients with stroke and their caregivers of the purpose and significance of the study, and asked them to sign the informed consent. The research assistants taught the participants how to self-complete the questionnaire; if a participant could not read the questionnaire, the research assistants interviewed the caregivers, read the questions for them and recorded their answer. The research assistants collected the questionnaires after the participants finished and verified with the caregivers that all of the recorded responses were correct. If caregivers were busy with taking care of patients and could not complete the questionnaire, we marked their questionnaire as invalid. We then randomly selected 40 caregivers from the 409 participants to complete a second questionnaire two weeks after the initial survey. We conducted the interviews of the caregivers between January 10, 2016 and August 3, 2016. We obtained the demographic and medical characteristics of patients with stroke and the demographic characteristics of stroke caregivers.

### Data analysis

Two research assistants coded and scored all participant responses, after which they then conducted two verifications of the responses and the scoring (S1 Dataset). We conducted our analysis using SPSS version 21.0 and Amos 17.0. For the principal variables, we produced descriptive statistics, including means, standard deviations, skewness and kurtosis.

We calculated the item total correlation using Pearson correlation analysis. The value of the item-total correlation was greater than 0.3 and statistical significance testing indicated a desirable discriminating power [37]. We divided extreme groups by 27% and 73% of the total score of AC-QoL, and conducted an extreme group comparison using independent-samples t-test. We estimated test-retest reliability using Pearson correlation analysis and the intraclass correlation coefficient (ICC) with a two-week interval between evaluations. We calculated the ICC estimates and their 95% confident intervals using SPSS and an absolute-agreement two-way mixed-effects model [38]. We used Cronbach’s alpha and a model-based internal consistency index (a measure used by previous researchers to estimate the reliability of the total multidimensional scale [39,40]) to evaluate the internal consistency reliability of our Chinese-language AC-QoL.

We calculated the I-CVI as the ratio of the number of expert-opinion “highly relevant” and “quite relevant” responses to the number of experts; we calculated the S-CVI as the average of the I-CVI for all the items rated as either “highly relevant” or “quite relevant” [41].

We used CFA to determine the goodness of fit of our sample data in the model proposed by Schreiber et al. [42]. We used the normal theory maximum likelihood estimation in CFA on all the item scores to confirm the eight factors in the original version [31]. In order to get the best-fitting structure and the appropriate number of factors in the Chinese version, we used the following criteria: 1) a value for the goodness-of-fit index (GFI) and the adjusted GFI (AGFI) of at least 0.9; 2) a root mean square error of approximation (RMSEA) of less than 0.06; 3) a non-significant goodness-of-fit chi-square test; 4) a comparative fit index (CFI) value greater than 0.9; 5) values of χ2/df between 1 and 2; and 6) values of Bentler and Bonett’s normed-fit index (NFI) between 0 and 1 and greater than 0.90 [43,44].

When a CFA fails to fit the factor structure in the original instrument, EFA can be used to improve the model [45]. We therefore used EFA with principal component extraction and Varimax rotation to identify the factor structure of the Chinese AC-QoL, according to the protocol used by researchers in a previous study [45].

### Ethical consideration

Permission to translate and use the AC-QoL into Chinese was granted by the original authors. We obtained permission to conduct this study from the Zhengzhou University ethical committee in China (S1 File). We obtained informed consent from the Director of the Hospitals, the head of the clinical unit and all study participants. Stroke survivors and their caregivers were informed of the purpose of the study and what would be expected of them. Participants were given assurances of refusal or withdrawal from the study without any negative consequences and signed the written informed consent. We assured the participants that their responses would be kept anonymous and confidential. Our program began in 2013, and obtained our ethical consent in 2013. We started translate the AC-QOL in 2015, and then performed our survey in 2016.

## Results

After excluding invalid questionnaires, our study population consisted of 409 stroke caregivers.The demographic and medical characteristics of the stroke patients are shown in Table 1. The stroke patients ranged in age from 22 years old to 97 years old and the average age was 64.13 years old (standard deviation [SD] 16.30). Most patients were male (64.10%), married (81.4%), and had finished primary education (33.3%), but were not employed (80%). Approximately 61.9% of the patients had health insurance at the city level. Most of the patients had an ischemic stroke (65.5%), for a majority of patients (63.3%) this was their first stroke (63.3%), and the majority of stroke events had occurred no more than 6 months previously (56.0%).

**Table 1:** Demographic and medical characteristics of stroke patients (n = 409).

Variable	Category	N (%)
**Age (years old)**	<40	32 (7.8)
	40–49	53 (13.0)
	50–59	74 (18.1)
	60–69	89 (21.8)
	70–79	71 (17.3)
	≥80	90 (22.0)
**Gender**	Male	262 (64.1)
	Female	147 (35.9)
**Marital status**	Married	333 (81.4)
	Single/divorced/widowed	76 (18.6)
**Education level**	Primary	135 (33.0)
	Secondary	114 (27.9)
	High school	89 (21.8)
	University	71(17.3)
**Household income per month (Yuan)**	1000∼	78 (19.1)
	2000∼	60 (14.7)
	3000∼	271 (66.2)
**Health insurance**	Province level	22 (5.4)
	City level	253 (61.8)
	New Rural Cooperative	78 (19.1)
	Others	56 (13.7)
**Work status**	Working staff	82 (20)
	Not employed	327 (80)
**Type of stroke**	Ischemic	267 (65.3)
	Hemorrhagic	88 (21.5)
	Mixed	54 (13.2)
**Stroke number**	1	259 (63.3)
	2	94 (23.0)
	3 or above	56 (13.7)
**Duration of the illness**	1–6 month	229 (56.0)
	7–12month	22 (5.4)
	≥13month	158 (38.6)

The demographic characteristics of stroke caregivers are shown in Table 2. The caregivers ranged in age from 18 years old to 83 years old and the average age was 48.92 years old (SD 16.30). Most caregivers were female (63.8%), married (87.3%), and had completed high school or a higher level of education (52.8%). Nearly half of the caregivers (47.7%) were the children of the stroke patients. The majority of caregivers did not live with the patients (75.6%), but provided care to them for more than 12 hours per day (64.1%), and were not employed (56.0%).

**Table 2:** Demographic characteristics of stroke caregivers (n = 409).

Variable	Category	N (%)
**Age (years old)**	<40	95 (23.3)
	40–49	97 (23.7)
	50–59	122 (29.8)
	60–69	70 (17.1)
	70–79	18 (4.4)
	≥80	7 (1.7)
**Gender**	Male	148 (36.2)
	Female	261 (63.8)
**Marital status**	Married	357 (87.3)
	Single/divorced/widowed	52 (12.7)
**Education level**	Primary	79 (19.3)
	Secondary	114 (27.9)
	High school	110(26.9)
	University	106 (25.9)
**Relationship**	Spouse	155 (37.9)
	Daughters/sons	195 (47.7)
	Parents	47 (11.5)
	Other relatives	12 (2.9)
**Live with patient**	Yes	309 (24.4)
	No	100 (75.6)
**Duration of caregiving care hours per day**	4~	75 (18.3)
	8~	72 (17.6)
	12h~	262 (64.1)
**Work status**	Working staff	180 (44.0)
	No job	229 (56.0)

We tested an 8-factor model, in keeping with the structure of the original AC-QoL version. The criteria to evaluate the model fit are shown in Table 3. Our results indicated that we needed to perform EFA to modify the model for the Chinese AC-QoL.

**Table 3:** Goodness-of-fit indices for the AC-QoL factor models.

Index	Stroke Caregivers
**Goodness of fit index (GFI)**	0.723
**Adjusted GFI (AGFI)**	0.682
**Chi-square**	3044.655
**Chi-square DF**	4.276
**Pr > Chi-square**	<0.001
**RMSEA estimate**	0.090
**RMSEA 90% lower confidence limit**	0.086
**RMSEA 90% upper confidence limit**	0.093
**Comparative fit index (CFI)**	0.867
**Normed-fit index (NFI)**	0.786

Our results indicated that the item total correlation values ranged from 0.317 to 0.734 (all with *p* < 0.001); the item total correlation value of item 19 was 0.193, and that of item 37 was 0.244, so we deleted these items before we performed EFA. Extreme group comparison *t* scores ranged from 5.501 to -17.976 (all with *p* < 0.05) and the test-retest reliability value was 0.86. The ICC value was 0.924 (95% confidence interval [CI] 0.857 to 0.960). The I-CVI values ranged from 0.90 to 1.0, and the S-CVI was 0.98.

**Fig 1:**
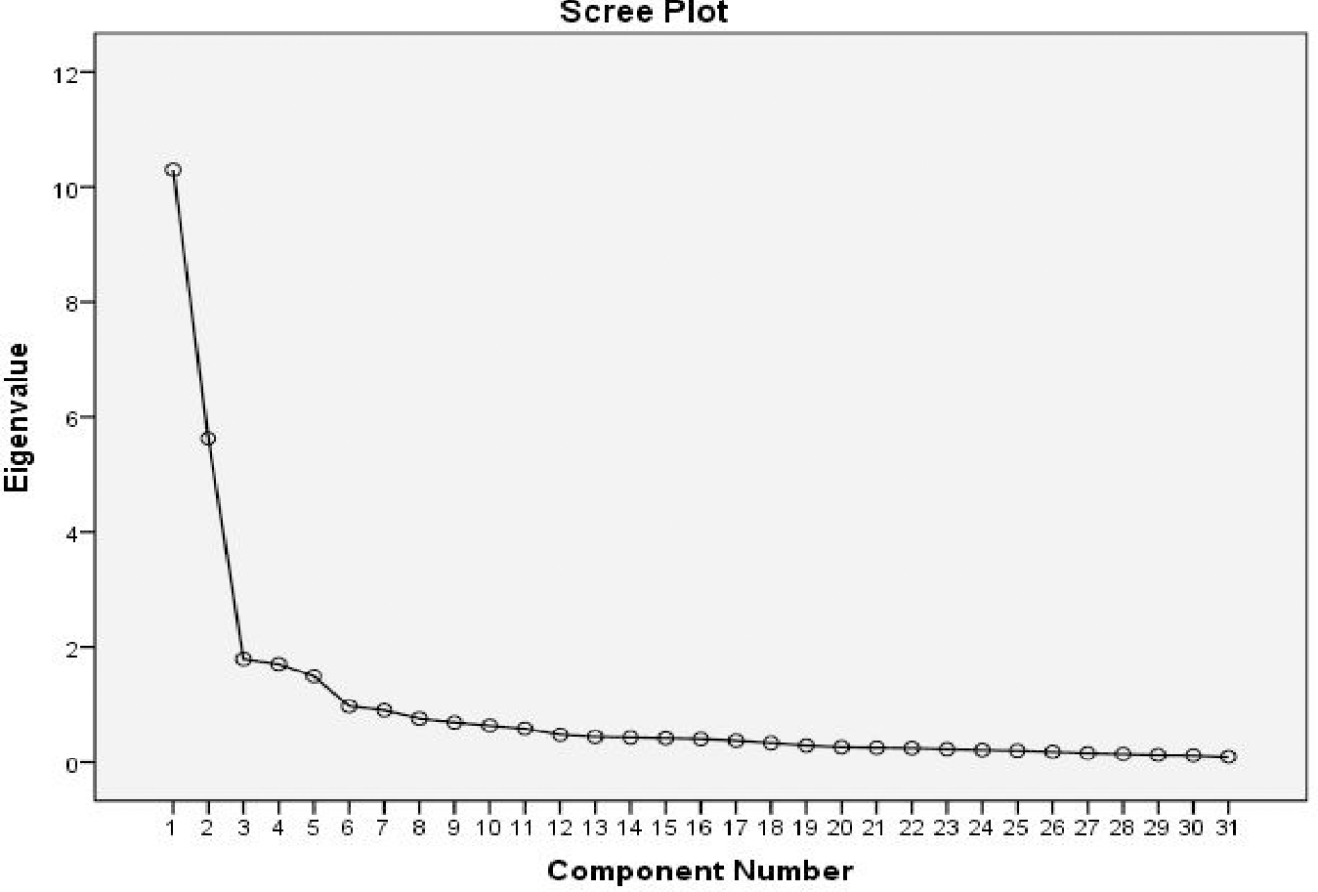
Scree plot of Chinese version AC-QoL. AC-QoL: Adult Carer Quality of Life Questionnaire.

There were five factors in the model, which we rotated using Varimax, accounting for 69.27% of the total variance (the details are shown in Table 4). Our estimates of the Kaiser-Meyer-Olkin measure of sampling adequacy (KMO) and Bartlett’s test indicated that our data was suitable for EFA (KMO = 0.908, χ2 = 9530.99, *P* < 0.001). We extracted five components with a variance of 67.56% according to the standard and each factor’s loading should be greater than 1 (scree plot shown in Fig 1). We considered for inclusion in the five components each item with a loading value that was higher than 0.4 (Table 4). Based on our EFA results, we named factor 1 “Caring Benefits”, while factors 2, 3, 4 and 5 were in the same domain as the original version, so we kept the same names (“Caring Stress”, “Caring Choice”, “Support for Caring” and “Money Matters”). We moved item 38 from “Carer Satisfaction” in the original version to “Caring Stress.” We removed items 1, 16, 19, 22, 24, 25, 28, 29, and 37 based on our predetermined criteria.

**Table 4:** Five factors with factor loading for stroke caregivers (n = 409).

Item	Factor Loading (FL)				
**Caring Benefits**	**Factor 1**				
34. I can manage most situations with the person I care for	0.874				
32. I can take care of the needs of the person I am caring for	0.827				
35. I am able to deal with a difficult situation	0.811				
30. I have a good relationship with the person I am caring for	0.806				
31. I am satisfied with my performance as a carer	0.784				
36. Caring is important to me	0.779				
33. I feel I am able to make the life of the person I am looking after better	0.776				
27. The person I look after respects me for what I do	0.723				
26. I feel valued by the person I am looking after	0.723				
40. I am satisfied with my life as a carer	0.712				
23. Because of caring, I feel that I have grown as a person	0.704				
39. I enjoy being a carer	0.638				
21. I have become a more tolerant person through my caring role	0.628				
**Caring Stress**		**Factor 2**			
12. I feel worn out as a result of caring		0.899			
13. I am mentally exhausted by caring		0.891			
14. I am physically exhausted by caring		0.865			
15. I feel stressed as a result of caring		0.793			
11. I feel depressed due to caring		0.762			
38. I feel frustrated with the person I am caring for		0.522			
**Caring Choice**			**Factor 3**		
8. I feel I have less choice about my future due to caring			0.832		
9. I feel I have no control over my own life			0.798		
10. Caring stops me doing what I want to do			0.780		
7. My social life has suffered because of caring			0.734		
6. I feel that my life is on hold because of caring			0.653		
**Support for Caring**				**Factor 4**	
3. I am happy with the professional support that is provided to me				0.780	
2. My needs as a carer are considered by professionals				0.770	
4. I feel able to get the help and information I need				0.768	
5. I have all the practical support I need				0.765	
**Money Matters**					**Factor 5**
17. I feel satisfied with my financial situation					0.852
18. I am able to save for a rainy day					0.803
20. There is enough money in our house to pay for the things we need					0.682
**Eigenvalue**	10.304	5.631	1.797	1.710	1.500
**Variance,%**	32.239	18.163	5.796	5.513	4.840
**Cumulative variance**	32.239	51.408	57.198	62.715	67.555

The Cronbach’s alpha of our Chinese-language AC-QoL for stroke caregivers was 0.924, Cronbach’s alpha for the subdomains ranged from 0.779–0.920, and the model-based reliability coefficient was 0.914. These results indicated that our instrument had good reliability.

Table 5 shows the descriptive statistics of our Chinese version of the AC-QoL (S2 File) in caregivers for stroke survivors. The overall mean score of caregivers on the AC-QoL was 55.20 with an SD of 15.88. Each item had low to moderate non-normality.

**Table 5:** The descriptive statistics of Chinese version AC-QoL in caregivers (n = 409).

Item	Mean	SD	Skewness	Kurtosis
**Factor 1 (Caring Benefits)**	25.32	9.04	-0.38	-0.62
34. I can manage most situations with the person I care for	1.99	0.80	-0.34	-0.54
32. I can take care of the needs of the person I am caring for	2.11	0.74	-0.36	-0.56
35. I am able to deal with a difficult situation	1.88	0.86	-0.27	-0.72
30. I have a good relationship with the person I am caring for	2.23	0.78	-0.64	-0.42
31. I am satisfied with my performance as a carer	1.97	0.84	-0.35	-0.68
36. Caring is important to me	2.18	0.82	-0.68	-0.31
33. I feel I am able to make the life of the person I am looking after better	2.05	0.81	-0.48	-0.43
27. The person I look after respects me for what I do	1.95	0.97	-0.45	-0.90
26. I feel valued by the person I am looking after	1.95	0.93	-0.41	-0.84
40. I am satisfied with my life as a carer	1.73	0.92	-0.08	-0.96
23. Because of caring, I feel that I have grown as a person	1.83	1.07	-0.37	-1.15
39. I enjoy being a carer	1.56	0.96	0.05	-0.97
21. I have become a more tolerant person through my caring role	1.88	0.99	-0.38	-0.98
**Factor 2 (Caring Stress)**	12.19	4.53	-0.78	-0.01
12. I feel worn out as a result of caring	2.00	0.92	-0.67	-0.36
13. I am mentally exhausted by caring	1.94	0.95	-0.61	-0.52
14. I am physically exhausted by caring	1.92	0.92	-0.55	-0.49
15. I feel stressed as a result of caring	1.83	0.89	-0.47	-0.44
11. I feel depressed due to caring	2.17	0.88	-0.87	0.00
38. I feel frustrated with the person I am caring for	2.31	0.85	-1.09	0.41
**Factor3 (Caring Choice)**	9.41	3.82	-0.56	-0.16
8. I feel I have less choice about my future due to caring	1.84	0.95	-0.32	-0.91
9. I feel I have no control over my own life	1.97	1.00	-0.65	-0.65
10. Caring stops me doing what I want to do	1.86	090	-0.44	-0.53
7. My social life has suffered because of caring	1.91	0.92	-0.48	-0.62
6. I feel that my life is on hold because of caring	1.83	0.89	-0.37	-0.59
**Factor4 (Support for Caring)**	4.15	3.58	0.84	-0.08
3 I am happy with the professional support that is provided to me	1.12	1.12	0.76	-0.80
2. My needs as a carer are considered by professionals	0.99	1.05	0.97	-0.23
4. I feel able to get the help and information I need	1.07	1.07	0.86	-0.50
5. I have all the practical support I need	0.97	1.03	1.01	-0.08
**Factor 5 (Money Matters)**	4.13	2.34	0.20	-0.49
17. I feel satisfied with my financial situation	1.23	0.97	0.27	-0.94
18. I am able to save for a rainy day	1.44	0.94	0.17	-0.86
20. There is enough money in our house to pay for the things we need	1.47	0.91	0.11	-0.77
Overall instrument	55.20	15.88	-0.24	-0.21

**SD:** Standard Deviations

## Discussion

Our work is the first to indicate a cultural variable factor structure in caregivers of patients with stroke in China. Our findings are similar to those of Ozer et al [45].

Interestingly, the results of our factor analysis suggested that some of the AC-QoL items are not intrinsic to the QoL of stroke caregivers. Nine items were not included in the factor structure of our Chinese version. This may be due to differences between the Chinese culture and the British culture. In a manner similar to the Chinese caregivers of cancer patients [24], our Chinese caregivers may have assumed that they would be the providers of care for their loved ones, whether parents or spouse. This may be related to the concept of filial piety in Chinese culture [46].

The characteristics of our stroke caregivers may also have influenced the differences between Chinese version and English version. Our caregivers were mostly younger than their counterparts in the UK. Reports from the literature indicate that advancing age in stroke caregivers puts those caregivers at an increased risk for adverse outcomes [47]. Our caregivers had also cared for their loved ones for significantly less time than their British counterparts (six months versus twelve years, on average) [31], and previous researchers have demonstrated that caregivers had lower burden and higher general self-efficacy in the first six months post-stroke [48]. In addition, the families of the stroke patients in our study were relatively well-off financially (most had a monthly income of more than 3000 Yuan), which may have influenced their responses to the questions, i.e., they may have felt less of a financial burden with respect to caregiving (in keeping with the findings of Hanratty et al. [49]), unlike caregivers from rural areas who are poor. Further exploration of these differences should be conducted in larger groups from different economic levels of society.

Our Chinese-language AC-QoL contained five domains (Caring Benefits, Caring Stress, Caring Choice, Support for Caring and Money Matters). This is similar to the Chinese version of the cancer caregiver QoL instrument, which (after cross-cultural adaptation) included domains for Burden, Disruptiveness, Positive Adaptation and Financial Concern [24]. According to White et al.’s model of QoL for family caregivers of stroke survivors [23], caring stress is a caregiver factor, whereas support for caring and money matters are environmental factors. Brand et al. demonstrated that more benefit finding by caregivers can increase their perceived QoL [11]. Thus, our Chinese version of AC-QoL covered a wide range of the aspects of QoL and focused on both the negative and the positive outcomes of caregivers.

There are a few limitations to the generalizability of our findings. First, we used a convenience sampling method and we recruited participants from hospitals only in Zhengzhou, China, which means our findings cannot be generalized to caregivers from other provinces. Second, we recruited our participants from within the hospital setting—the QoL of caregivers may be significantly different within their communities and their homes. Future researchers should recruit participants from communities. Third, we did not evaluate a criterion-related validation of the constructs in our Chinese version of the AC-QoL. Research should be conducted on the criterion-related validation using well-developed tools such as the SF-36 or WHOQOL. The validity assessment is needed in future research.

Despite these limitations, our findings indicate that our version of the AC-QoL is a reliable and valid tool which can be used to assess the QoL of stroke caregivers in mainland China, which may have some important implications. First, health care providers should consider the QoL of caregivers for stroke patients, which they can assess using the AC-QoL. Second, the AC-QoL may be useful as a potential screening tool to detect caregivers who need help. Third, assessing both the positive and negative aspects of the QoL of stroke caregivers provides a more comprehensive view of caregivers, which can help us to understand better the caregiving experience and caregivers themselves. Fourth, we can investigate more deeply the factors influencing the QoL of caregivers, including factors which may mediate or modify the interrelating domains of QoL. Fifth, the AC-QoL can be used to assess the effectiveness of interventions, which may help to improve the QoL of caregivers. Moreover, an improved understanding of the QoL of caregivers may help us understand how the QoL of caregivers may affect that of stroke patients. A better understanding of the association of caregiver factors with patient outcomes may have implications for rehabilitation research, professional practice, policy directions and resource allocation [50]. Finally, QoL can be implemented as a cost-effective measure in studies of interventions attempting to improve the outcomes of stroke survivors [51]. Reports from the literature indicate that the QoL of stroke survivors is highly correlated with that of their caregivers, and is greatly associated with the physical function changes of stroke survivors [16]. The findings from previous studies indicate that caregiver training is associated with less financial burden and a higher QoL for both stroke survivors and their caregivers [52, 53]. However, there is limited evidence that interventions for caregivers of stroke survivors are (cost) effective, thus more economic evaluations are needed [53].

In conclusion, this study provides some evidence on the reliability and validity of the AC-QoL is a reliable and valid tool which can be used to assess the QoL of stroke caregivers in mainland China. Our version of the AC-QoL has slightly different items and components when compared with the English version. We therefore suggest that instruments from developed countries may need to be modified when used in developing countries or in different cultures.

## Supporting information

S1 File Ethic statement. (PDF)

S2 File Chinese version of Adult Carer Quality of Life questionnaire. (PDF)

S1 Dataset Dataset for data analysis. (SAV)
