# Supplementary material for: Validity and reliability of Chinese version of Adult Carer Quality of Life questionnaire (AC-QoL) in family caregivers of stroke survivors
Source: PLoS One. 2017 Nov 13;12(11):e0186680. doi: 10.1371/journal.pone.0186680 (PMC5683601; doi:10.1371/journal.pone.0186680)
Supplement: S1 File — (PDF) [file pone.0186680.s001.pdf]

郑州大学生命科学伦理审查委员会

伦 理 审 查 报 告

项目名称： 脑卒中患者连续康复护理规范与流程研究（134200510018）

Title ： The research on standard of continuous rehabilitation for stroke patients（13420051001）

项目负责人： 张振香（Zhenxiang Zhang）

伦理审查意见：

该研究将研究目的及方法告知研究对象，得到了患者及照顾者的知情同意。

经郑州大学生命科学伦理审查委员会审查，该项目研究内容和过程遵循国际及国家颁布的有关生物医学研究的伦理要求，同意该项目实施。

Ethical review:

The patients and caregivers learn the aim and research protocol, and informed consent will be obtained from all patients and caregivers in this study.

This study was investigated by institute’s Internal Review Board（IRB）or Ethics Committee of Zhengzhou University, China, and the approval has been granted.

郑州大学生命科学伦理审查委员会

Ethics Committee of Zhengzhou University

2013 年 3 月 14 日

郑州大学生命科学伦理审查委员会

伦 理 审 查 报 告

项目名称： 脑卒中患者连续康复护理规范与流程研究（134200510018）

Title ： The research on standard of continuous rehabilitation for stroke patients（13420051001）

项目负责人： 张振香（Zhenxiang Zhang）

伦理审查意见：

该研究将研究目的及方法告知研究对象，得到了患者及照顾者的知情同意。

经郑州大学生命科学伦理审查委员会审查，该项目研究内容和过程遵循国际及国家颁布的有关生物医学研究的伦理要求，同意该项目实施。

Ethical review:

The patients with stroke and their caregivers learned the aims and research protocol, and signed the informed consents in this study.

This study was investigated by institute’s Internal Review Board（IRB）or Ethics Committee of Zhengzhou University, China, and the approval has been granted.

郑州大学生命科学伦理审查委员会

Ethics Committee of Zhengzhou University

2016 年 1 月 5 日
