# Supplementary material for: Validity and reliability of Chinese version of Adult Carer Quality of Life questionnaire (AC-QoL) in family caregivers of stroke survivors
Source: PLoS One. 2017 Nov 13;12(11):e0186680. doi: 10.1371/journal.pone.0186680 (PMC5683601; doi:10.1371/journal.pone.0186680)
Supplement: S2 File — (PDF) [file pone.0186680.s002.pdf]

成人照顾者生活质量问卷

问卷填写指导语：此部分主要是要了解您作为照顾者生活不同方面的情况，请回顾一下您最近两周作为照顾者的感受并选择出每一条与您相符的状况。答案没有对错，我们主要想了解您作为照顾者的生活情况。请真实的回答每个问题，在适当的方框内划“√”。

	从来没有	有时	大部分时间	总是
1. 我能与患者共同应对多数境况				
2. 我能顾及患者的需求				
3 我能应对困境				
4. 我与患者关系融洽				
5. 我满意自己作为照顾者的表现				
6 .照顾患者对我很重要				
7. 我觉得我能使患者的生活更美好				
8. 患者尊重我为他做的一切				
9. 我感到了我对患者的价值				
10. 我满意自己作为照顾者的生活				
11. 我觉得照顾患者使我自身成长了				
12. 我能享受成为一个照顾者				
13. 照顾患者让我更加宽容				
14. 照顾患者使我感到筋疲力竭				
15. 照顾患者使我精神疲乏				
16. 照顾患者使我身体疲惫				
17. 我感受到了照顾患者的压力				
18. 照顾患者使我感到郁闷				
19. 照顾患者令我感到沮丧				
20. 我觉得照顾患者减少了我对今后的选择				
21. 我觉得无法控制自己的生活				
22. 照顾患者妨碍了我做自己想做的事				
23. 照顾患者打乱了我的社交生活				
24. 我觉得照顾患者限制了我的生活				
25. 我满意提供给我的专业支持				
26. 我作为照顾者的需求得到了专业人员的关注				
27. 我觉得我能获得需要的帮助和信息				
28. 我获得了我需要的实际支持				
29. 我满意我的经济状况				
30. 我能存些钱以备急用				
31. 我们有购买所需物品的一定财力				

For English version: http://dx.doi.org/10.1108/13619321211270380
